# Primary parotid lymphoepithelial carcinoma: A case report and literature review of a rare pathological entity

**DOI:** 10.1016/j.ijscr.2020.06.035

**Published:** 2020-06-12

**Authors:** Ashley Whelan, Ahmed A. Al-Sayed, Martin Bullock, S. Mark Taylor

**Affiliations:** a Faculty of Medicine, Dalhousie University, 5849 University Ave, Halifax, NS B3H 4R2, Canada; b Division of Otolaryngology Head and Neck Surgery, Department of Surgery, Dalhousie University, 5850 University Avenue, Halifax, NS B3K 6R8, Canada; c Department of Otolaryngology Head and Neck Surgery, King Saud University, King Abdul Aziz Rd, Al Malaz, Riyadh 12629, Saudi Arabia; d Department of Pathology, Dalhousie University, Sir Charles Tupper Medical Building, Room 11B, 5850 College Street, Halifax, NS B3H 4R2, Canada

**Keywords:** LEC, lymphoepithelial carcinoma, NPC, nasopharyngeal carcinoma, EBV, Ebstein-Barr virus, CT, computed tomography, MRI, magnetic resonance imaging, FNA, fine needle aspiration, H&E, Hematoxylin & Eosin stain, cm, centimeters, Lymphoepithelial carcinoma, Malignant salivary gland neoplasms, Malignant parotid neoplasms, EBV neoplasms

## Abstract

**Background:**

Lymphoepithelial carcinoma (LEC) of the salivary glands is a rare, but distinct, poorly-differentiated neoplasm that resembles undifferentiated nasopharyngeal carcinomas (NPC). Among primary salivary gland LECs, they most commonly arise in the parotid gland. These lesions have a noticeable racial predilection, mostly occurring in Asians and Arctic region native populations. They are strongly associated with Epstein-Barr virus (EBV), and are more common in EBV-endemic areas. The most common presenting symptoms of primary parotid LEC are parotid mass development and cervical lymphadenopathy. We report an unusual case of EBV-negative LEC in a Canadian Caucasian woman.

**Case presentation:**

A 40-year-old female from Atlantic Canada presented with a rapidly enlarging preauricular mass and cervical lymphadenopathy. Preoperative diagnostics via radiology and fine needle aspiration alluded to an ambiguous primary parotid malignancy. She underwent a total parotidectomy with facial nerve sacrifice and ipsilateral selective neck dissection. The facial nerve was reconstructed with a lateral antecubital nerve graft. Pathology came back positive for LEC, and she began adjuvant radiotherapy. At her 1-year follow up, she was disease-free and obtained a House-Brackmann facial nerve function grade of 3.

**Conclusions:**

The case report highlights the importance of having a high index of suspicion and the need for interdisciplinary collaboration in reaching the diagnosis of primary parotid LEC. LEC can afflict patients of any ethnicity in non-EBV endemic areas and should therefore be considered in all patients with a painless parotid mass regardless of ethnicity. Further studies are required to elucidate the oncogenic role of EBV in these cancers.

## Highlights

• A high index of suspicion is required for primary parotid lymphoepithelial cancer in Caucasians with a pre-auricular mass.
• LEC can afflict patients of any ethnicity in Epstein-Barr Virus non-endemic areas.
• LEC of the parotid gland is a rare entity that requires complete surgical resection and post-operative radiation therapy.

## 1 Background

Lymphoepithelial carcinomas (LEC) are rare poorly-differentiated neoplasms of epithelial cells that are characterized by extensive lymphoid infiltration in the stroma. They are histopathological analogues to undifferentiated nasopharyngeal carcinomas (NPC) and most commonly occur in the nasopharynx. However, LEC has been reported to arise primarily in various organs such as lungs, stomach, breast, ovaries, uterus, renal pelvis, bladder and skin [15,20]. In addition to the nasopharynx, within the head and neck region, LECs can arise in salivary glands, tonsils, thymus, larynx, and soft palate [15,20]. Primary LEC of the salivary gland, first described by Hilderman et al. in 1962, is very rare and accounts for 0.4% of all malignant tumors of the salivary glands [1,2]. 82% of LEC cases of the head and neck occur in either the parotid gland or submandibular gland with a 7:1 ratio [2].

It is important to distinguish between primary LEC of the salivary glands from secondary metastatic NPC to the salivary glands as the treatment for each differs. Similar to NPC, primary LEC also has a very strong racial and geographical predilection, predominantly affecting the Asian, and Arctic circle native populations, with less than 15% of all primary parotid LECs involving patients of Caucasian descent [2]. Like NPC, primary LEC has a strong association with Epstein-Barr virus (EBV) particularly in EBV-endemic areas [2]. Interestingly, for LEC of salivary glands, this association is less consistent in non-EBV endemic regions, and in lower-risk ethnic populations suggesting a complex interaction between environmental and hereditary factors [21]. Because LEC and NPC are histologically indistinguishable, imaging with nasopharyngeal CT or nasopharyngoscopy, with or without random nasopharyngeal biopsies are often performed to rule out metastatic disease.

Clinically, the most common presenting symptoms of LEC of the parotid gland is a non-tender parotid mass of variable duration, although pain or discomfort may be present. Approximately 20% of cases present with concurrent facial nerve palsy, and 40% with cervical lymphadenopathy, both ipsilateral to the primary lesion. [2]. The rate of regional lymph node metastasis of LEC varies but has been reported up to 40% [3], and some studies have found that 20% of patients develop distant metastatic lesions within 3 years of being treated [16]. Common locations of distant metastases include the lungs, liver, brain, and bone. The median age of diagnosis for parotid gland LEC is 40 years, and there is an overall 3:2 female preponderance [2] except in Chinese populations where these tumors appear to be more common in men [9]. Reports of LEC in the Western literature are very limited, with only 17 cases reported in Caucasian patients and variability in EBV status. We report an unusual case of EBV-negative LEC in a Caucasian woman from Canada in line with the SCARE criteria [22]. Our aim is to shed the light on primary LEC as a differential diagnosis in patients presenting with a parotid mass with multiple clinical and/or radiological lymphadenopathy without any obvious nasopharyngeal primary malignancy—regardless of their ethnicity and EBV status.

## 2 Case report

**Fig. 1:**
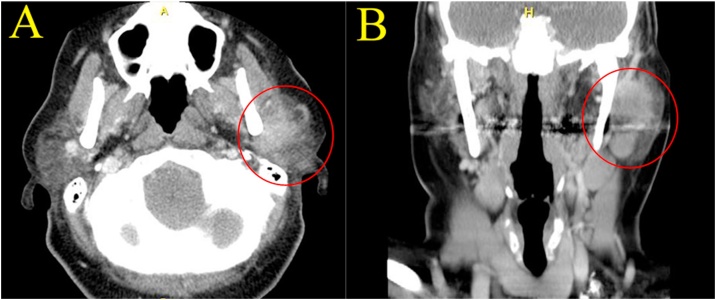
Computed tomography images: A. Axial slice showing a heterogeneously enhancing left parotid mass with deep lobe involvement B. Coronal reconstruction showing the same enhancing mass.

A 40-year-old Caucasian female from Prince Edward Island, Canada, presented for evaluation of a left preauricular mass of two years’ duration. The patient indicated that the mass had recently seemed firmer and she had become more aware of its presence in the few months leading up to presentation. From a symptomatology perspective, the patient denied pain or tenderness, and hadn’t noticed any facial weakness on the involved side. Her past medical history included a remote sleeve gastrectomy due to obesity, an ankle injury repair, and a cholecystectomy. She was not on any medications. She did not smoke or consume alcohol and had no known allergies. Her family history was negative for head and neck malignancies. On physical examination, a rubbery, fixed, 5-centimeter (cm) nontender mass was easily palpable in the left parotid. She did not have any palpable cervical lymph nodes. Facial nerve function was unremarkable. Flexible nasopharyngoscopy was unremarkable. An initial fine needle aspiration (FNA) was done on the parotid mass at a satellite head and neck clinic on Prince Edward Island, revealing atypical cells. Imaging was also requested, and a computed tomography (CT) scan demonstrated a heterogeneously enhancing solid mass in the left parotid, measuring 3.5 cm × 3.2 cm × 4.6 cm with a component of deep lobe extension and ill-defined margins. Multiple pathological lymph nodes were also seen along the vicinity of the mass and in the neck (Fig. 1). A chest x-ray was done with unremarkable findings. A second FNA was performed in Halifax, Nova Scotia with the hope of a better diagnostic yield. The results indicated the presence of malignant cells, consistent with a poorly differentiated carcinoma.

**Fig. 2:**
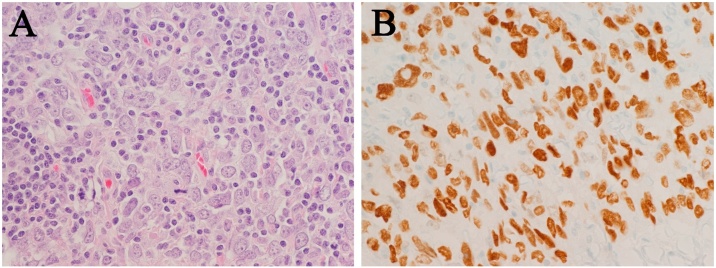
**A**) Photomicrograph showing discohesive high grade tumors cells with irregular nuclei and prominent nucleoli. There are no light microscopic features of squamous or glandular differentiation (eg. intercellular bridges, keratinization or mucin production) but the cells stained positively for broad spectrum keratin, consistent with a carcinoma. There are small mature lymphocytes in the background (H&E 400×). **B**) Immunohistochemistry for the p63 antibody, consistent with squamous differentiation (400×).

As such, she was scheduled for surgery. A total parotidectomy with facial nerve sacrifice and ipsilateral selective neck dissection were performed. Intraoperatively, the mass was found adherent to surrounding structures, encasing vessels, and also the main trunk of the facial nerve. This required resection of the main nerve trunk along with a component of the upper and lower divisions, while preserving the peripheral branches via retrograde dissection, and ligation of the external carotid. A lateral antecubital nerve graft was used to anastomose the resected temporal and buccal facial nerve segments to the main trunk. All frozen section margins of the proximal and distal facial nerve were negative. The mass was submitted enbloc and an ipsilateral neck dissection of levels II and III was performed without any issues. On microscopic evaluation, however, there was extraparotid extension with margin involvement. There was also extensive neurovascular involvement, including perineural and intraneural invasion, as well as angioinvasion. Multiple intraglandular lymph nodes came back positive, as well as neck nodes from levels II and III, with one node demonstrating extranodal extension. The mass was determined to be a poorly differentiated carcinoma, consistent with a primary lymphoepithelial carcinoma of the parotid on final histopathology. Microscopically, the tumor showed a syncytial growth pattern of undifferentiated, high-grade malignant cells with a patchy infiltrate of small lymphocytes in the background. No glandular or squamous differentiation was distinguished on hematoxylin and eosin sections. (Fig. 2). However, on immunohistochemistry, the neoplastic cells were cytokeratin 8/18, high molecular weight keratin and p63 positive, consistent with a carcinoma. The presence of p63 positivity is indicative of squamous differentiation, which is typical of many lymphoepithelial carcinomas, as well as undifferentiated nasopharyngeal carcinoma. The presence of EBV in the pathological specimen was tested by in situ hybridization for EBV-encoded RNA; however, it was found to be negative. There was no evidence of a pre-existing pleomorphic adenoma.

She was referred for radiotherapy for the primary site and the neck basin, and then followed up on an annual basis. One year following surgery, she had no evidence of recurrence and was able to close her eye completely. She obtained a House-Brackmann grade 3 out of 6 for her facial nerve function. At the five year follow up mark, she remained recurrence free at the primary site, but unfortunately developed multiple pulmonary metastases and received chemotherapy.

## 3 Discussion

Lymphoepithelial carcinoma of the salivary glands is a distinct and rare tumor, closely associated to anaplastic carcinoma, poorly differentiated carcinoma, or undifferentiated carcinoma. LECs of the head and neck primarily involve either the parotid gland or submandibular gland at a ratio of 7:1 [2]. Microscopically, they typically manifest as islands of large, spindle-shaped or epithelioid cells with large vesicular nuclei containing one or more prominent nucleoli, in a benign lymphoid background [2]. As these tumors have both epithelial and lymphoid components, similar to benign lymphoepithelial lesions, some authors have used terms such as “undifferentiated carcinoma with lymphoid stroma” or “carcinoma ex lymphoepithelial lesion”. In this case, there was no evidence of a background benign lymphoepithelial lesion, and the infiltrate of small lymphocytes could be considered a reactive “tumor-associated lymphoid proliferation”. Similarly, high grade salivary carcinomas of many types may arise from a pre-existing pleomorphic adenoma (carcinoma ex pleomorphic adenoma), but in this case there was no evidence of a pre-existing adenoma.

These tumors have a noticeable racial predilection, mostly occurring in Asians and Arctic region natives. They are uncommon among Americans and Europeans, with Caucasians comprising less than 15% of reported cases [2]. Furthermore, there’s a strong association with EBV, and hence they have a higher incidence rate in EBV-endemic areas. However, in non-endemic areas the association is less consistent, brining into question the oncogenic role of EBV in these neoplasms [2]. To date, there have only been 17 reported cases of LEC in Caucasian patients (Table 1).

**Table 1:** Clinicopathological features of parotid LEC cases reported in the Western literature.

Author	Gender	Age	Treatment	EBV status	Clinical status
Ferlito A, 1977 [4]	Female	36	SP	N/A	At the last follow-up, no signs of the disease
Ferlito A, 1977 [4]	Female	55	SP	N/A	At the last follow-up, no signs of the disease
Ferlito A, 1977 [4]	Female	32	CP	N/A	At the last follow-up, no signs of the disease
Kott ET, 1984 [5]	Male	51	RT	N/A	At 11-year follow-up, no signs of the disease
Kott ET, 1984 [5]	Female	42	SP + RT	N/A	At 12-month follow-up, no signs of the disease
Kountakis Se, 1995 [6]	Female	68	SP + RT	Negative	At 24-month follow-up, no signs of the disease
Kountakis Se, 1995 [6]	Female	57	CP + RT	Negative	At 24-month follow-up, no signs of the disease
Kotsianti A, 1996 [7]	Male	64	SP	Positive	At the last follow-up, no signs of the disease
Squillaci S, 2000 [8]	Female	45	SP + RT	Positive	At 15-month follow-up, no signs of the disease
Wu DL, 2001 [9]	Female	54	CP + RT	Positive	At 24-month follow-up, no signs of the disease
Squillaci S, 2002 [10]	Female	72	SP + RT	Positive	At 36-month follow-up, no signs of the disease
Bialas M, 2002 [11]	Female	74	RT	Positive	N/A
Ayache S, 2004 [12]	Male	47	CP + RT	Negative	At 7-month follow-up, no signs of the disease
Manganaris A, 2007 [13]	Female	67	SP + RT	N/A	At 12-month follow-up, no signs of the disease
Abdelkrim S, 2009 [14]	Female	70	CP	Negative	At 5-months, no signs of the disease
Ambrosio M, 2013 [15]	Female	45	CP	Positive	At 20-months, no signs of the disease
Salcedo-Gil C, 2019 [16]	Male	59	Accessory parotid gland excision	Negative	At 3.5-year follow up, no signs of the disease

EBV, *Ebstein-Barr Virus,* SP *Superficial Parotidectomy,* CP *Complete Parotidectomy,* RT *Radiation Therapy,* N/A *Not Available.*

Clinically, the average age at presentation is typically during the fourth to fifth decade of life. This neoplasm affects more females than males with a ratio of approximately 3:2 [2], except in Chinese populations where they have been reported more often in males [9]. The most common presenting symptoms are salivary gland swelling that is often painless, and cervical lymphadenopathy. They typically exhibit an indolent growth rate often for the first few years of their appearance followed by an accelerated increase in size in the months prior to their clinical presentation. The facial nerve is usually not affected at presentation but can be affected in up to 20% of cases [2]. The most common site of metastasis is the cervical lymph nodes (41.3%) with distant metastases often involve the lung, liver, bone, and brain [2]. Routine laboratory investigations are noncontributory [2].

FNA is a safe and relatively low-cost procedure used to detect malignancy of the salivary glands [24,25]. While the sensitivity and specificity of FNA is lower than core needle biopsy (CNB) [25,26], the use of a smaller-bore needle in FNA is associated with decreased risk of tumor seeding, injury to the facial nerve, and hematoma [24,[27], [28], [29]]. In a retrospective review of ultrasound-guided needle biopsy of parotid lesions, Romano et al. (2017) showed that FNA with selective CNB in cases where preliminary cytopathology with the FNA specimen alone cannot yield a definitive diagnosis, produces a favorable balance between diagnostic accuracy and risk of complications associated with CNB [24]. FNA in our patient yielded a conclusive cytopathological result and a CNB was therefore not pursued. Although FNAs might allow early detection of malignant cells, the diagnostic accuracy of FNAs in LEC specifically was found to be 78.6% in a recent review of 14 patients with LEC [17].

Given the limited diagnostic accuracy of FNA, radiological imaging is a valuable tool in the preoperative evaluation of parotid LEC. Ban et al. (2014) found that most parotid LEC present on CT and MRI as poorly defined masses with lobulated or plaque-like appearance. These lesions typically show homogeneous signal intensity on unenhanced CT and MRI, with no signs of cystic degeneration or calcification. Absence of tumor necrosis may also be diagnostically useful as most salivary gland tumors lack necrotic regions. These radiological features in conjunction with clinical presentation, FNA results, and an absence of nasopharyngeal lesions on nasopharyngoscopy/nasopharyngeal CT and/or biopsy suggests a diagnosis of parotid LEC.

Treatment modalities for LEC of the salivary glands include surgical excision, radiation therapy, and chemotherapy [2]. Establishing the correct diagnosis is of paramount importance in choosing the ideal treatment modality. In general, due to the similarities between LEC and NPC in the same ethnic populations, the strong association with EBV, histopathology, and immunohistochemistry, a clinical distinction must be made between primary and secondary (metastatic) LEC. Therefore, the need for a meticulous fiberoptic nasopharyngoscopy or nasopharyngeal CT should be emphasized. At present, surgical excision followed by postoperative adjuvant radiation therapy is considered the treatment of choice for primary LEC of the salivary gland. Tang et al. (2012) proposed three possible indications for a total parotidectomy versus superficial parotidectomy including: a tumor involving the deep lobe of the parotid gland, preoperative FNA showing high-grade malignancy, and a relatively large tumor. Postoperative radiotherapy is considered mainstay in the treatment of salivary gland LEC due to their radiosensitivity [3]. The 40% risk of metastasis to regional lymph nodes necessitates consideration for an ipsilateral selective neck dissection depending on cervical lymph node involvement. Although the facial nerve is clinically intact on presentation 80% of the time, parotid LEC often invades the nerve necessitating resection and reconstruction to limit the inherent morbidity of complete surgical resection.

The introduction of microsurgery and other techniques made the practice of facial nerve resection in the setting of parotid malignancy when the nerve is uninvolved completely unacceptable [23]. The facial nerve function greatly affects the patient’s quality of life and should be preserved whenever possible. However, in our reported case, the facial nerve was invaded by the carcinoma, and nerve preservation was not possible without compromising oncologic safety. In such situations when the sacrifice of the involved portion of the facial nerve is necessary, immediate reconstruction for function restoration is necessary to limit the morbidity and improve quality of life. Multiple options exist for reconstruction. Immediate reconstruction with nerve interposition grafting is often the most preferred method. It can be combined with other dynamic or static facial reanimation procedures. We opted to reconstruct the facial with an interposition graft using the great auricular nerve in a single stage procedure given that intraoperative frozen sections showed complete resection with negative margins, the resected portion was small, and avoiding a donor site and another procedure was possible. Alternatively, facial nerve reconstruction can be planned in multiple stages. This could prove necessary when the resected segment is large and/or occurs proximally at the stylomastoid foramen.

Because of the rarity of this neoplasm, it is difficult to formulate prognostic and survival data. Some reports suggest that LEC has a better prognosis than other undifferentiated carcinomas of the salivary glands, in part due to their radiosensitivity, and because the lymphoid stroma is thought to have a role in limiting the aggressiveness of the tumor [2]. Local recurrence rates have been reported up to 28.9% [18], and approximately 20% of patients develop distant metastasis within 3 years of being treated [16]. However, with combined surgical excision and postoperative radiotherapy, the 2, 5, and 10 year survival rates are reported to be 91%, 66%, and 29%, respectively [19].

## 4 Conclusion

LEC of the parotid gland is a rare malignant neoplasm that requires complete surgical resection of the primary tumor, ipsilateral selective neck dissection, and postoperative adjuvant radiation therapy. The facial nerve, if involved, should be resected and primarily reconstructed to avoid morbidity. Patient evaluation should include a proper history and careful physical examination including fiberoptic nasopharyngoscopy or nasopharyngeal CT to rule out NPC clinically before the diagnosis of LEC is accepted. FNA of the lesion should be obtained as part of the evaluation and repeated when in doubt or if the results are inconclusive. Imaging in the form of CT and/or MRI are useful diagnostic aids and should be obtained as part of the preoperative diagnosis and planning. Rarely, as is highlighted in this case, it can affect Caucasians in non-EBV endemic areas, illustrating the need to consider primary parotid LEC even in ethnically and demographically low-risk patients. Therefore, a high index of suspicion and interdisciplinary collaboration are required to reach the diagnosis. Moreover, this case report highlights the need for further studies that are required to elucidate the oncogenic role of EBV in these cancers.

## Declaration of Competing Interest

The authors declare that they have no competing interests.

## Sources of funding

No funding was required for this case report.

## Ethics approval and consent to participate

This case report was performed under the Nova Scotia Health Authority Research Ethics Board guidelines for case reports. No formal research ethics board approval was necessary and therefore no reference number was generated.

## Consent

Written informed consent was obtained from the patient for publication of this case report and any accompanying images. A copy of the written consent is available for review by the Editor-in-Chief of this journal.

## Author contribution

**Ashley Whelan:** investigation and writing (original draft).

**Ahmed A. Al-Sayed:** investigation, validation and writing (original draft).

**Martin Bullock:** formal analysis and writing (review and editing).

**S. Mark Taylor:** conceptualization, supervision, and writing (review and editing).

## Registration of research studies

1. Name of the registry: N/A.
2. Unique identifying number or registration ID: N/A.
3. Hyperlink to your specific registration (must be publicly accessible and will be checked): N/A.

## Guarantor

Ashley Whelan.

## Availability of data and material

Data sharing is not applicable to this article as no datasets were generated or analysed during the current study. Data sharing is unavailable for this study as it would compromise patient privacy. However, further information regarding the case is available, within limits of patient privacy, upon request.

## Provenance and peer review

Not commissioned, externally peer-reviewed.
